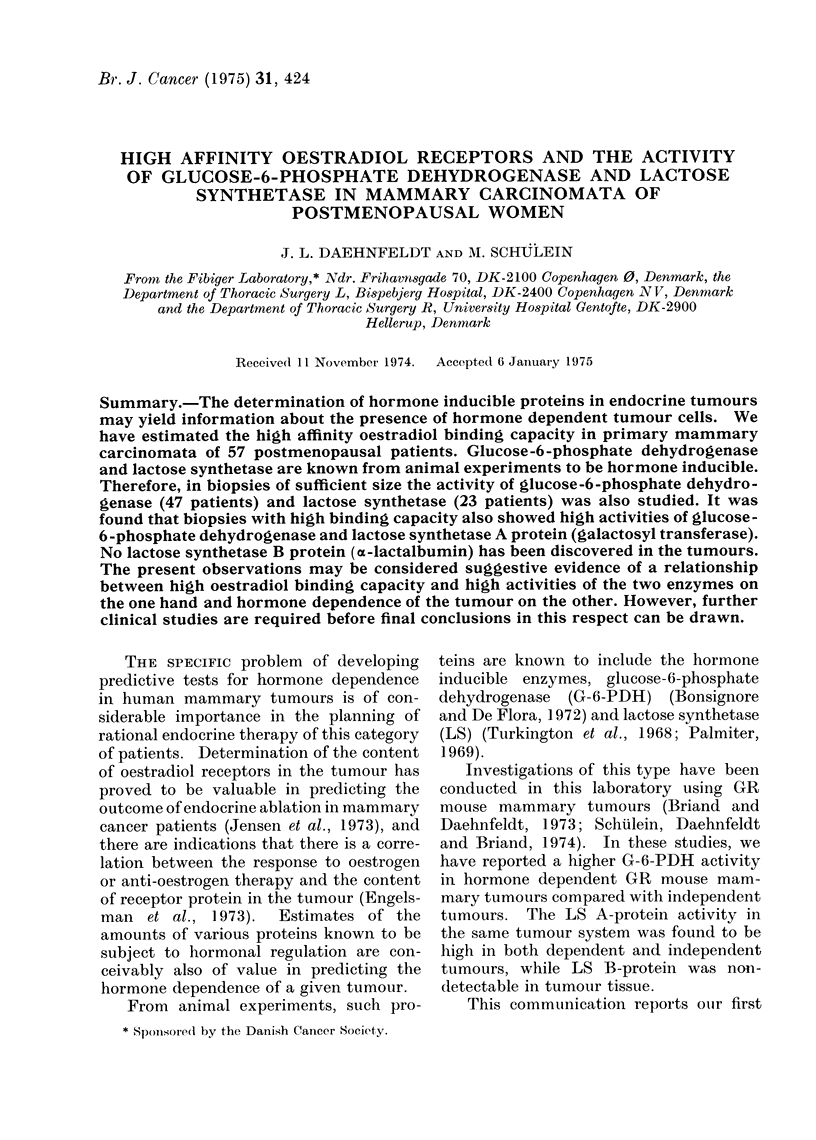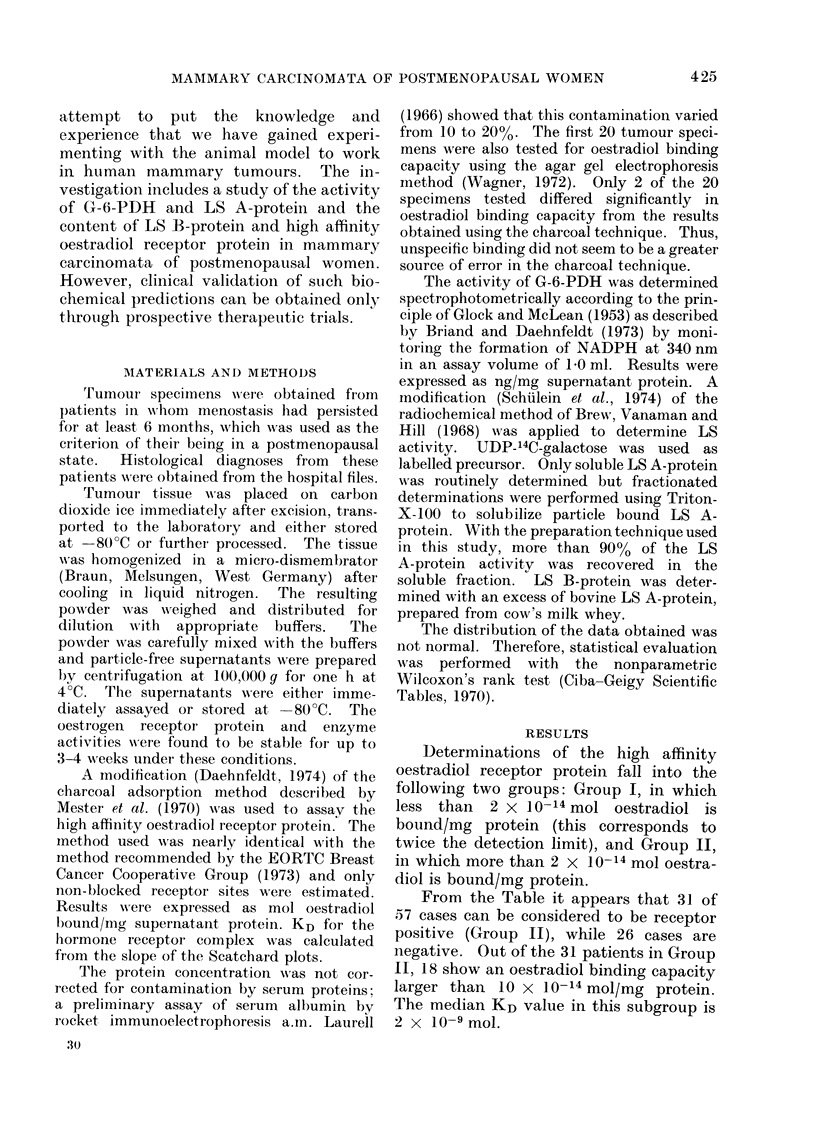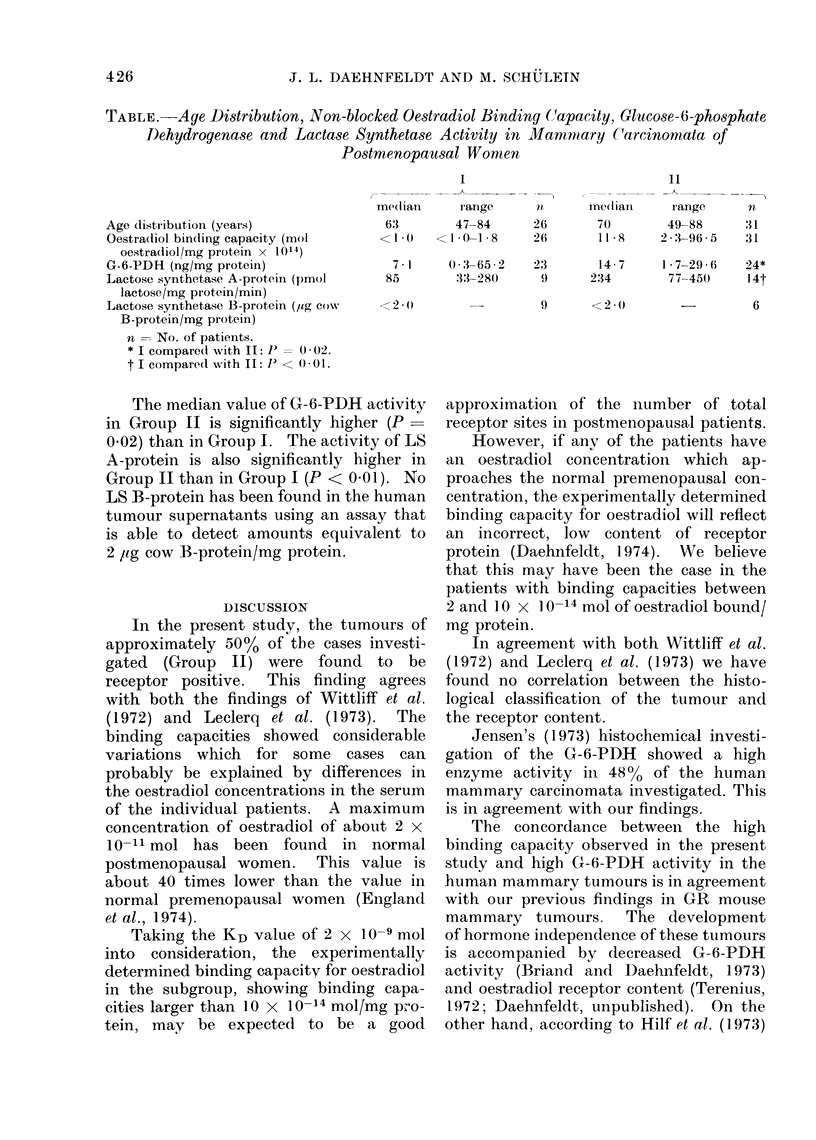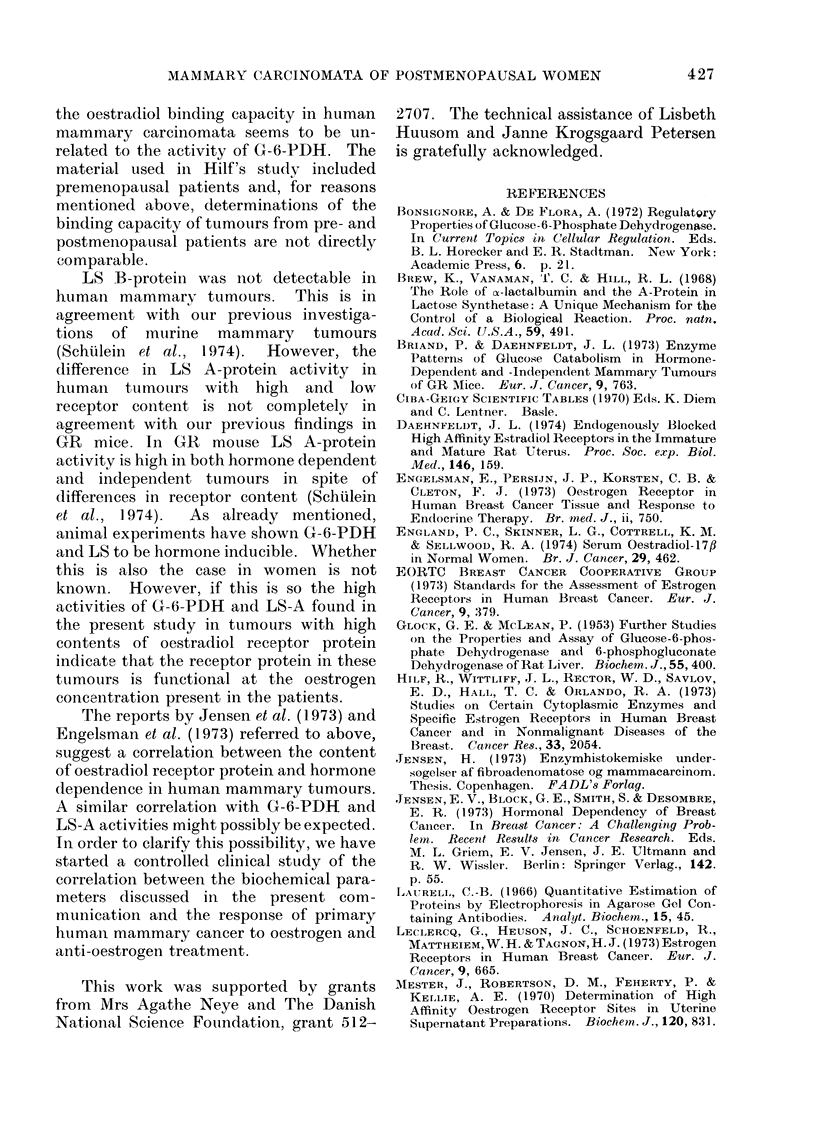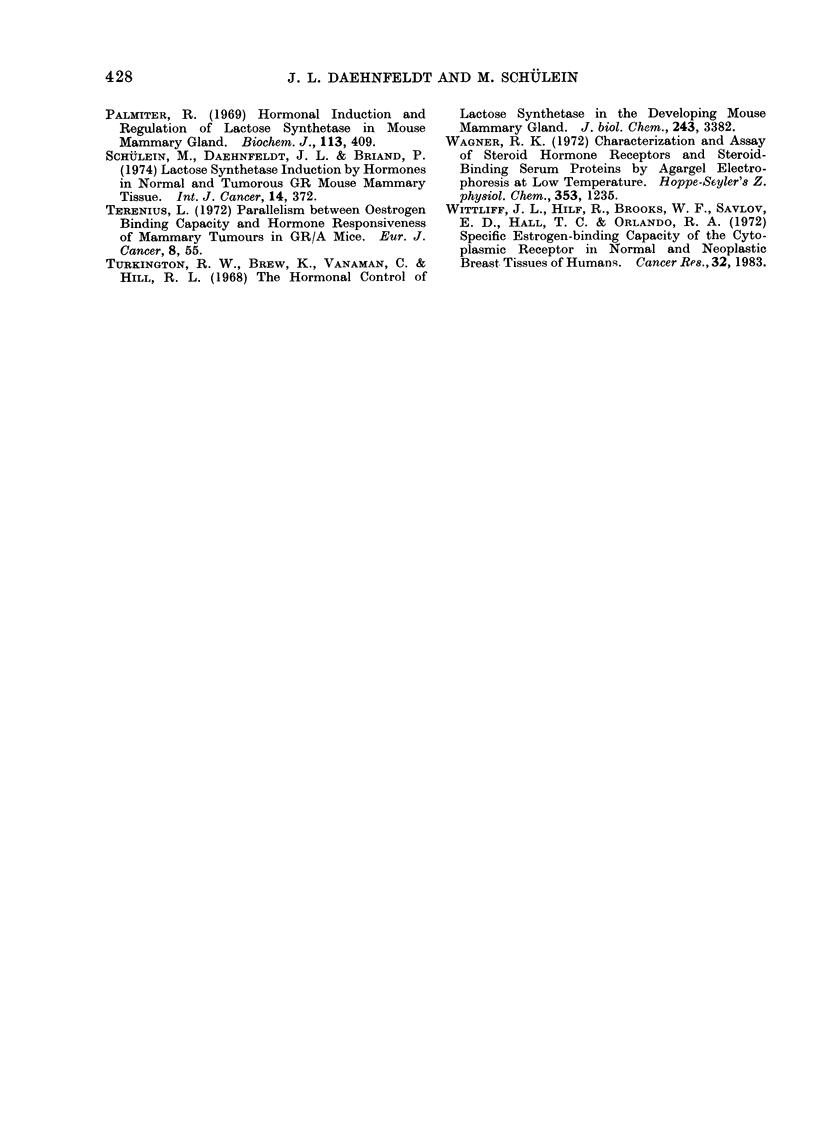# High affinity oestradiol receptors and the activity of glucose-6-phosphate dehydrogenase and lactose synthetase in mammary carcinomata of postmenopausal women.

**DOI:** 10.1038/bjc.1975.81

**Published:** 1975-04

**Authors:** J. L. Daehnfeldt, M. Schülein

## Abstract

The determination of hormone inducible proteins in endocrine tumours may yield information about the presence of hormone dependent tumour cells. We have estimated the high affinity oestradiol binding capacity in primary mammary carcinomata of 57 postmenopausal patients. Glucose-6-phosphate dehydrogenase and lactose synthetase are known from animal experiments to be hormone inducible. Therefore, in biopsies of sufficient size the activity of glucose-6-phosphate dehydrogenase (47 pateints) and lactose synthetase (23 patients) was also studied. It was found that biopsies with high binding capacity also showed high activities of glucose-6-phosphate dehydrogenase and lactose synthetase A protein (galactosyl transferase). No lactose synthetase B protein (alpha-lactalbumin) has been discovered in the tumours. The present observations may be considered suggestive evidence of a relationship between high oestradiol binding capcity and high activities of the two enzymes on the one hand and hormone dependence of the tumour on the other. However, further clinical studies are required before final conclusions in this respect can be drawn.


*Br. J. Cancer* (1975) **31**, 424

### HIGH AFFINITY OESTRADIOL RECEPTORS AND THE ACTIVITY OF GLUCOSE-6-PHOSPHATE DEHYDROGENASE AND LACTOSE SYNTHETASE IN MAMMARY CARCINOMATA OF POSTMENOPAUSAL WOMEN

J. L. DAEHNFELDT AND M. SCHÜLEIN

*From the Fibiger Laboratory,* Ndr. Frihavnsgade 70, DK-2100 Copenhagen Ø, Denmark, the Department of Thoracic Surgery L, Bispebjerg Hospital, DK-2400 Copenhagen NV, Denmark and the Department of Thoracic Surgery R, University Hospital Gentofte, DK-2900 Hellerup, Denmark*

Received 11 November 1974. Accepted 6 January 1975

**Summary.—The determination of hormone inducible proteins in endocrine tumours may yield information about the presence of hormone dependent tumour cells. We have estimated the high affinity oestradiol binding capacity in primary mammary carcinomata of 57 postmenopausal patients. Glucose-6-phosphate dehydrogenase and lactose synthetase are known from animal experiments to be hormone inducible. Therefore, in biopsies of sufficient size the activity of glucose-6-phosphate dehydrogenase (47 patients) and lactose synthetase (23 patients) was also studied. It was found that biopsies with high binding capacity also showed high activities of glucose-6-phosphate dehydrogenase and lactose synthetase A protein (galactosyl transferase). No lactose synthetase B protein (α-lactalbumin) has been discovered in the tumours. The present observations may be considered suggestive evidence of a relationship between high oestradiol binding capacity and high activities of the two enzymes on the one hand and hormone dependence of the tumour on the other. However, further clinical studies are required before final conclusions in this respect can be drawn.**

THE SPECIFIC problem of developing predictive tests for hormone dependence in human mammary tumours is of considerable importance in the planning of rational endocrine therapy of this category of patients. Determination of the content of oestradiol receptors in the tumour has proved to be valuable in predicting the outcome of endocrine ablation in mammary cancer patients (Jensen *et al.*, 1973), and there are indications that there is a correlation between the response to oestrogen or anti-oestrogen therapy and the content of receptor protein in the tumour (Engelsman *et al.*, 1973). Estimates of the amounts of various proteins known to be subject to hormonal regulation are conceivably also of value in predicting the hormone dependence of a given tumour.

From animal experiments, such proteins are known to include the hormone inducible enzymes, glucose-6-phosphate dehydrogenase (G-6-PDH) (Bonsignore and De Flora, 1972) and lactose synthetase (LS) (Turkington *et al.*, 1968; Palmiter, 1969).

Investigations of this type have been conducted in this laboratory using GR mouse mammary tumours (Briand and Daehnfeldt, 1973; Schülein, Daehnfeldt and Briand, 1974). In these studies, we have reported a higher G-6-PDH activity in hormone dependent GR mouse mammary tumours compared with independent tumours. The LS A-protein activity in the same tumour system was found to be high in both dependent and independent tumours, while LS B-protein was nondetectable in tumour tissue.

This communication reports our first

* Sponsored by the Danish Cancer Society.

attempt to put the knowledge and experience that we have gained experimenting with the animal model to work in human mammary tumours. The investigation includes a study of the activity of G-6-PDH and LS A-protein and the content of LS B-protein and high affinity oestradiol receptor protein in mammary carcinomata of postmenopausal women. However, clinical validation of such biochemical predictions can be obtained only through prospective therapeutic trials.

### MATERIALS AND METHODS

Tumour specimens were obtained from patients in whom menostasis had persisted for at least 6 months, which was used as the criterion of their being in a postmenopausal state. Histological diagnoses from these patients were obtained from the hospital files.

Tumour tissue was placed on carbon dioxide ice immediately after excision, transported to the laboratory and either stored at −80°C or further processed. The tissue was homogenized in a micro-dismembrator (Braun, Melsungen, West Germany) after cooling in liquid nitrogen. The resulting powder was weighed and distributed for dilution with appropriate buffers. The powder was carefully mixed with the buffers and particle-free supernatants were prepared by centrifugation at 100,000 *g* for one h at 4°C. The supernatants were either immediately assayed or stored at −80°C. The oestrogen receptor protein and enzyme activities were found to be stable for up to 3–4 weeks under these conditions.

A modification (Daehnfeldt, 1974) of the charcoal adsorption method described by Mester *et al.* (1970) was used to assay the high affinity oestradiol receptor protein. The method used was nearly identical with the method recommended by the EORTC Breast Cancer Cooperative Group (1973) and only non-blocked receptor sites were estimated. Results were expressed as mol oestradiol bound/mg supernatant protein. $K_D$ for the hormone receptor complex was calculated from the slope of the Scatchard plots.

The protein concentration was not corrected for contamination by serum proteins; a preliminary assay of serum albumin by rocket immunoelectrophoresis a.m. Laurell (1966) showed that this contamination varied from 10 to 20%. The first 20 tumour specimens were also tested for oestradiol binding capacity using the agar gel electrophoresis method (Wagner, 1972). Only 2 of the 20 specimens tested differed significantly in oestradiol binding capacity from the results obtained using the charcoal technique. Thus, unspecific binding did not seem to be a greater source of error in the charcoal technique.

The activity of G-6-PDH was determined spectrophotometrically according to the principle of Glock and McLean (1953) as described by Briand and Daehnfeldt (1973) by monitoring the formation of NADPH at 340 nm in an assay volume of 1·0 ml. Results were expressed as ng/mg supernatant protein. A modification (Schülein *et al.*, 1974) of the radiochemical method of Brew, Vanaman and Hill (1968) was applied to determine LS activity. UDP-$^{14}$C-galactose was used as labelled precursor. Only soluble LS A-protein was routinely determined but fractionated determinations were performed using Triton-X-100 to solubilize particle bound LS A-protein. With the preparation technique used in this study, more than 90% of the LS A-protein activity was recovered in the soluble fraction. LS B-protein was determined with an excess of bovine LS A-protein, prepared from cow's milk whey.

The distribution of the data obtained was not normal. Therefore, statistical evaluation was performed with the nonparametric Wilcoxon's rank test (Ciba–Geigy Scientific Tables, 1970).

### RESULTS

Determinations of the high affinity oestradiol receptor protein fall into the following two groups: Group I, in which less than $2 \times 10^{-14}$ mol oestradiol is bound/mg protein (this corresponds to twice the detection limit), and Group II, in which more than $2 \times 10^{-14}$ mol oestradiol is bound/mg protein.

From the Table it appears that 31 of 57 cases can be considered to be receptor positive (Group II), while 26 cases are negative. Out of the 31 patients in Group II, 18 show an oestradiol binding capacity larger than $10 \times 10^{-14}$ mol/mg protein. The median $K_D$ value in this subgroup is $2 \times 10^{-9}$ mol.

TABLE.—*Age Distribution, Non-blocked Oestradiol Binding Capacity, Glucose-6-phosphate Dehydrogenase and Lactase Synthetase Activity in Mammary Carcinomata of Postmenopausal Women*

| | I | | | II | | |
|---|---|---|---|---|---|---|
| | median | range | $n$ | median | range | $n$ |
| Age distribution (years) | 63 | 47–84 | 26 | 70 | 49–88 | 31 |
| Oestradiol binding capacity (mol oestradiol/mg protein $\times 10^{14}$) | $<1 \cdot 0$ | $<1 \cdot 0$–$1 \cdot 8$ | 26 | 11·8 | 2·3–96·5 | 31 |
| G-6-PDH (ng/mg protein) | 7·1 | 0·3–65·2 | 23 | 14·7 | 1·7–29·6 | 24* |
| Lactose synthetase A-protein (pmol lactose/mg protein/min) | 85 | 33–280 | 9 | 234 | 77–450 | 14† |
| Lactose synthetase B-protein ($\mu$g cow B-protein/mg protein) | $<2 \cdot 0$ | — | 9 | $<2 \cdot 0$ | — | 6 |

$n$ = No. of patients.

* I compared with II: $P = 0 \cdot 02$.

† I compared with II: $P < 0 \cdot 01$.

The median value of G-6-PDH activity in Group II is significantly higher ($P = 0{\cdot}02$) than in Group I. The activity of LS A-protein is also significantly higher in Group II than in Group I ($P < 0{\cdot}01$). No LS B-protein has been found in the human tumour supernatants using an assay that is able to detect amounts equivalent to 2 $\mu$g cow B-protein/mg protein.

### DISCUSSION

In the present study, the tumours of approximately 50% of the cases investigated (Group II) were found to be receptor positive. This finding agrees with both the findings of Wittliff *et al.* (1972) and Leclerq *et al.* (1973). The binding capacities showed considerable variations which for some cases can probably be explained by differences in the oestradiol concentrations in the serum of the individual patients. A maximum concentration of oestradiol of about $2 \times 10^{-11}$ mol has been found in normal postmenopausal women. This value is about 40 times lower than the value in normal premenopausal women (England *et al.*, 1974).

Taking the $K_D$ value of $2 \times 10^{-9}$ mol into consideration, the experimentally determined binding capacity for oestradiol in the subgroup, showing binding capacities larger than $10 \times 10^{-14}$ mol/mg protein, may be expected to be a good approximation of the number of total receptor sites in postmenopausal patients.

However, if any of the patients have an oestradiol concentration which approaches the normal premenopausal concentration, the experimentally determined binding capacity for oestradiol will reflect an incorrect, low content of receptor protein (Daehnfeldt, 1974). We believe that this may have been the case in the patients with binding capacities between 2 and $10 \times 10^{-14}$ mol of oestradiol bound/mg protein.

In agreement with both Wittliff *et al.* (1972) and Leclerq *et al.* (1973) we have found no correlation between the histological classification of the tumour and the receptor content.

Jensen's (1973) histochemical investigation of the G-6-PDH showed a high enzyme activity in 48% of the human mammary carcinomata investigated. This is in agreement with our findings.

The concordance between the high binding capacity observed in the present study and high G-6-PDH activity in the human mammary tumours is in agreement with our previous findings in GR mouse mammary tumours. The development of hormone independence of these tumours is accompanied by decreased G-6-PDH activity (Briand and Daehnfeldt, 1973) and oestradiol receptor content (Terenius, 1972; Daehnfeldt, unpublished). On the other hand, according to Hilf *et al.* (1973)

the oestradiol binding capacity in human mammary carcinomata seems to be unrelated to the activity of G-6-PDH. The material used in Hilf's study included premenopausal patients and, for reasons mentioned above, determinations of the binding capacity of tumours from pre- and postmenopausal patients are not directly comparable.

LS B-protein was not detectable in human mammary tumours. This is in agreement with our previous investigations of murine mammary tumours (Schülein *et al.*, 1974). However, the difference in LS A-protein activity in human tumours with high and low receptor content is not completely in agreement with our previous findings in GR mice. In GR mouse LS A-protein activity is high in both hormone dependent and independent tumours in spite of differences in receptor content (Schülein *et al.*, 1974). As already mentioned, animal experiments have shown G-6-PDH and LS to be hormone inducible. Whether this is also the case in women is not known. However, if this is so the high activities of G-6-PDH and LS-A found in the present study in tumours with high contents of oestradiol receptor protein indicate that the receptor protein in these tumours is functional at the oestrogen concentration present in the patients.

The reports by Jensen *et al.* (1973) and Engelsman *et al.* (1973) referred to above, suggest a correlation between the content of oestradiol receptor protein and hormone dependence in human mammary tumours. A similar correlation with G-6-PDH and LS-A activities might possibly be expected. In order to clarify this possibility, we have started a controlled clinical study of the correlation between the biochemical parameters discussed in the present communication and the response of primary human mammary cancer to oestrogen and anti-oestrogen treatment.

This work was supported by grants from Mrs Agathe Neye and The Danish National Science Foundation, grant 512–2707. The technical assistance of Lisbeth Huusom and Janne Krogsgaard Petersen is gratefully acknowledged.

### REFERENCES

Bonsignore, A. & De Flora, A. (1972) Regulatory Properties of Glucose-6-Phosphate Dehydrogenase. In *Current Topics in Cellular Regulation.* Eds. B. L. Horecker and E. R. Stadtman. New York: Academic Press, **6**. p. 21.

Brew, K., Vanaman, T. C. & Hill, R. L. (1968) The Role of α-lactalbumin and the A-Protein in Lactose Synthetase: A Unique Mechanism for the Control of a Biological Reaction. *Proc. natn. Acad. Sci. U.S.A.*, **59**, 491.

Briand, P. & Daehnfeldt, J. L. (1973) Enzyme Patterns of Glucose Catabolism in Hormone-Dependent and -Independent Mammary Tumours of GR Mice. *Eur. J. Cancer*, **9**, 763.

Ciba-Geigy Scientific Tables (1970) Eds. K. Diem and C. Lentner. Basle.

Daehnfeldt, J. L. (1974) Endogenously Blocked High Affinity Estradiol Receptors in the Immature and Mature Rat Uterus. *Proc. Soc. exp. Biol. Med.*, **146**, 159.

Engelsman, E., Persijn, J. P., Korsten, C. B. & Cleton, F. J. (1973) Oestrogen Receptor in Human Breast Cancer Tissue and Response to Endocrine Therapy. *Br. med. J.*, ii, 750.

England, P. C., Skinner, L. G., Cottrell, K. M. & Sellwood, R. A. (1974) Serum Oestradiol-17β in Normal Women. *Br. J. Cancer*, **29**, 462.

EORTC Breast Cancer Cooperative Group (1973) Standards for the Assessment of Estrogen Receptors in Human Breast Cancer. *Eur. J. Cancer*, **9**, 379.

Glock, G. E. & McLean, P. (1953) Further Studies on the Properties and Assay of Glucose-6-phosphate Dehydrogenase and 6-phosphogluconate Dehydrogenase of Rat Liver. *Biochem. J.*, **55**, 400.

Hilf, R., Wittliff, J. L., Rector, W. D., Savlov, E. D., Hall, T. C. & Orlando, R. A. (1973) Studies on Certain Cytoplasmic Enzymes and Specific Estrogen Receptors in Human Breast Cancer and in Nonmalignant Diseases of the Breast. *Cancer Res.*, **33**, 2054.

Jensen, H. (1973) Enzymhistokemiske undersøgelser af fibroadenomatose og mammacarcinom. Thesis. Copenhagen. *FADL's Forlag.*

Jensen, E. V., Block, G. E., Smith, S. & Desombre, E. R. (1973) Hormonal Dependency of Breast Cancer. In *Breast Cancer: A Challenging Problem. Recent Results in Cancer Research.* Eds. M. L. Griem, E. V. Jensen, J. E. Ultmann and R. W. Wissler. Berlin: Springer Verlag., **142**. p. 55.

Laurell, C.-B. (1966) Quantitative Estimation of Proteins by Electrophoresis in Agarose Gel Containing Antibodies. *Analyt. Biochem.*, **15**, 45.

Leclercq, G., Heuson, J. C., Schoenfeld, R., Mattheiem, W. H. & Tagnon, H. J. (1973) Estrogen Receptors in Human Breast Cancer. *Eur. J. Cancer*, **9**, 665.

Mester, J., Robertson, D. M., Feherty, P. & Kellie, A. E. (1970) Determination of High Affinity Oestrogen Receptor Sites in Uterine Supernatant Preparations. *Biochem. J.*, **120**, 831.

PALMITER, R. (1969) Hormonal Induction and Regulation of Lactose Synthetase in Mouse Mammary Gland. *Biochem. J.*, **113**, 409.

SCHÜLEIN, M., DAEHNFELDT, J. L. & BRIAND, P. (1974) Lactose Synthetase Induction by Hormones in Normal and Tumorous GR Mouse Mammary Tissue. *Int. J. Cancer*, **14**, 372.

TERENIUS, L. (1972) Parallelism between Oestrogen Binding Capacity and Hormone Responsiveness of Mammary Tumours in GR/A Mice. *Eur. J. Cancer*, **8**, 55.

TURKINGTON, R. W., BREW, K., VANAMAN, C. & HILL, R. L. (1968) The Hormonal Control of Lactose Synthetase in the Developing Mouse Mammary Gland. *J. biol. Chem.*, **243**, 3382.

WAGNER, R. K. (1972) Characterization and Assay of Steroid Hormone Receptors and Steroid-Binding Serum Proteins by Agargel Electrophoresis at Low Temperature. *Hoppe-Seyler's Z. physiol. Chem.*, **353**, 1235.

WITTLIFF, J. L., HILF, R., BROOKS, W. F., SAVLOV, E. D., HALL, T. C. & ORLANDO, R. A. (1972) Specific Estrogen-binding Capacity of the Cytoplasmic Receptor in Normal and Neoplastic Breast Tissues of Humans. *Cancer Res.*, **32**, 1983.